# Spur cells in liver cirrhosis are predictive of acute-on-chronic liver failure and liver-related mortality regardless of severe anaemia

**DOI:** 10.1007/s11739-023-03303-x

**Published:** 2023-05-22

**Authors:** Michele Bevilacqua, Leonardo De Marco, Roberta Stupia, Francesco Dima, Filippo Cattazzo, Veronica Paon, Donatella Ieluzzi, Andrea Dalbeni, David Sacerdoti

**Affiliations:** 1 Department of Medicine, General Medicine C, University of Verona, Azienda Ospedaliera Universitaria Integrata Verona, Verona, Italy; 2 Liver Unit, Department of Medicine, University and Azienda Ospedaliera Universitaria Integrata of Verona, Verona, Italy; 3 Clinical Biochemistry, University of Verona, Verona, Italy

**Keywords:** Spur cell anaemia, Liver cirrhosis, Hepatic decompensation, Liver-related mortality, ACLF, Liver transplantation

## Abstract

Chronic anaemia in advanced liver disease is a frequent finding. The aim was to explore the clinical impact of spur cell anaemia, a rare entity typically associated with end-stage of the disease. One-hundred and nineteen patients (73.9% males) with liver cirrhosis of any etiology were included. Patients with bone marrow diseases, nutrients deficiencies and hepatocellular carcinoma were excluded. In all patients, a blood sample was collected to check for the presence of spur cells on blood smear. A complete blood biochemical panel was recorded together with Child–Pugh (CP) score and Model for End-Stage Liver Disease (MELD) score. For each patients, clinically relevant events, such as acute-on-chronic liver failure (ACLF) and 1 year liver-related mortality, were registered. Patients were then grouped according to the percentage of spur cells at smear (> 5%, 1–5%, < 1%). Severe anaemia was defined as haemoglobin levels lower than 8 g/dL. 9.2% of subjects had > 5% spur cells, only 2 had evidence of haemolysis. In patients with > 5% spur cells, haemoglobin and albumin were lower compared with the other sub-group, while MELD score, CP score, International Normalized Ratio, ferritin, creatinine and unconjugated bilirubin were higher. Patients with more spur cells were more decompensated and developed more frequently ACLF. ACLF and liver-related mortality were significantly and independently associated with the presence of > 5% spur cells but not with baseline severe anaemia. Cirrhotic patients have a fairly high prevalence of spur cells, not always associated with severe haemolytic anaemia. The presence of spur red cells is per se associated with a worse prognosis and, therefore, should be always evaluated to prioritize patients for intensive management and eventually liver transplantation.

## Lay Summary

Chronic anaemia is a frequent finding in liver cirrhosis and spur cell anaemia has been shown to be an uncommon non-immune haemolytic disease typically related to advanced-liver disease. In our study, spur cell anaemia (spur cells >5%) was found in almost 10% of outpatient cirrhotics and was significantly related to more decompensated disease, higher incidence of ACLF and 1 year liver-related mortality. More importantly, the vast majority of patients with high percentage of spur cells did not have severe anaemia. Therefore, spur cells should be searched for in patients with advanced liver disease by a simple blood smear evaluation, even in the absence of significant anaemia, because of relevant prognostic impact and in order to prioritize patients to intensive management and possibly liver transplantation.

## Introduction

Chronic anaemia, (defined by World Health Organization when haemoglobin levels are < 13 g/dL in males, < 12 g/dL in non-pregnant females), is very common in liver disease and often represents a clinical challenge because of multifactorial etiologies [1, 2]. Malnutrition, malabsorption and dysmetabolism of vitamins (folates, vitamin B6, vitamin B12) and trace elements (iron, copper, selenium, manganese) are common in advanced liver cirrhosis, and need to be considered in chronic anaemia. Furthermore, chronic bleeding, due to portal hypertensive gastro-enteropathy, thrombocytopenia and severe coagulopathy, needs to be extensively evaluated. Erythropoietin deficiency of end-stage kidney disease, hematologic malignancy and bone marrow suppression due to alcohol, toxins, heavy metals together with various types of haemolysis (autoimmune [3], microangiopathic, copper-related in Wilson disease [4], Zieve syndrome in alcoholic hepatitis [5]) represent other possible factors [6–8]. Anaemia in liver cirrhosis is increasingly recognized as an important factor contributing to and predicting hepatic decompensation and mortality[9]. In this context, *spur cell anaemia* is a rare form of non-immune haemolytic anaemia in which red blood cells (RBC) are spiky-like and, thus, more prone to crack in splenic sinusoids. The disease is typically associated with alcoholic cirrhosis but the relationship between etiology and pathogenesis is poorly understood [10]. Only few studies have evaluated prevalence, risk factors and clinical impact of spur cell anaemia [11–13] which is almost always related to severe refractory haemolysis, advanced liver disease and poor outcome [14]. The only etiological therapy is liver transplantation (LT) [15]. Furthermore, it has been suggested that prognosis worsens significantly only when > 5% spur cells in blood smear are detected [11, 12]. Nevertheless, few data exist about the clinical significance of spur cells in blood smear when unrelated to overt haemolysis.

A case of severe refractory spur cell anaemia with unfortunate outcome is firstly presented.

Subsequently, the results of the prospective study are shown. The aim was to assess the prevalence of spur cells in blood smear in a cohort of outpatient cirrhotic patients and to search for clinical implications, such as acute-on-chronic liver failure (ACLF).

### Case report

A 71-year-old man recently diagnosed with alcoholic liver cirrhosis was referred to our Liver Unit for acute hepatic decompensation. Three months earlier, the patient had been hospitalized for ascites, sepsis, acute kidney injury and severe anaemia, and he was treated with antibiotics, renal replacement therapy and RBC transfusions. Chronic anaemia of unknown aetiology (no signs of gastro-intestinal bleeding, hematologic disorders, malignancy or iron/vitamins deficiency) was reported in recent medical history. The patient also previously tested negative for genetic hemochromatosis in marked hyperferritinemia (> 1500 µg/L) with 55% transferrin saturation. When admitted to hospital, he was febrile, anaemic, icteric, slightly confused and had tense ascites and peripheral oedema; however, he was hemodynamically stable (blood pressure 95/60 mmHg, pulse rate 80/min), showed satisfactory blood oxygen saturation in room air, and had impressive splenomegaly (20 cm diameter). As shown in Table 1, blood tests revealed severe macrocytic anaemia, leucocytosis, acute kidney injury, hypoalbuminemia, hyperbilirubinemia and severe coagulopathy (Child–Pugh score C). The patient was diagnosed with grade 2 ACLF (two organ failures: coagulopathy and severe acute kidney injury); biochemical analysis of ascitic fluid showed spontaneous bacterial peritonitis (white blood cells 6100/mm^3^, 83% neutrophils). He was treated with intravenous antibiotics, albumin and terlipressin, after diagnosing hepato-renal syndrome, with resolution of peritonitis and slow but progressive improvement of renal function.

**Table 1:** Laboratory values of the patient

Lab values (normal reference)	
Haemoglobin, g/dL (13,5–16,0)	6,0
Mean corpuscular volume, fL (80,0–99,0)	104
Platelets, *10^9/L (150 – 400)	50
Reticulocytes, % (0,50–2,10)	5,25
Total bilirubin, mg/dL (< 1,05]	8,49
Conjugated bilirubin, mg/dL (< 0,35)	3,31
Creatinine, mg/dL (0,8–1,2)	3,8
Haptoglobin, g/L (0,30–2,00)	< 0,08
INR (0,80–1,17)	2,4
Albumin, g/L (35–50)	30
Ferritin µg/L (20–200)	1482
Lactic dehydrogenase, U/L (135 –225)	264
Total cholesterol, mg/dL (100–200)	52
LDL cholesterol, mg/dL (50–130)	11
HDL cholesterol, mg/dL (40–80)	29
Triglycerides, mg/dL (80–150)	49
Apo-A1, g/L (1,10–2,05)	0,34
Apo-B, g/L (0,55–1,40)	0,24

*INR* International normalized ratio, *LDL* Low-density lipoprotein, *HDL* High-density lipoprotein, *Apo* Apolipoprotein

**Fig. 1:**
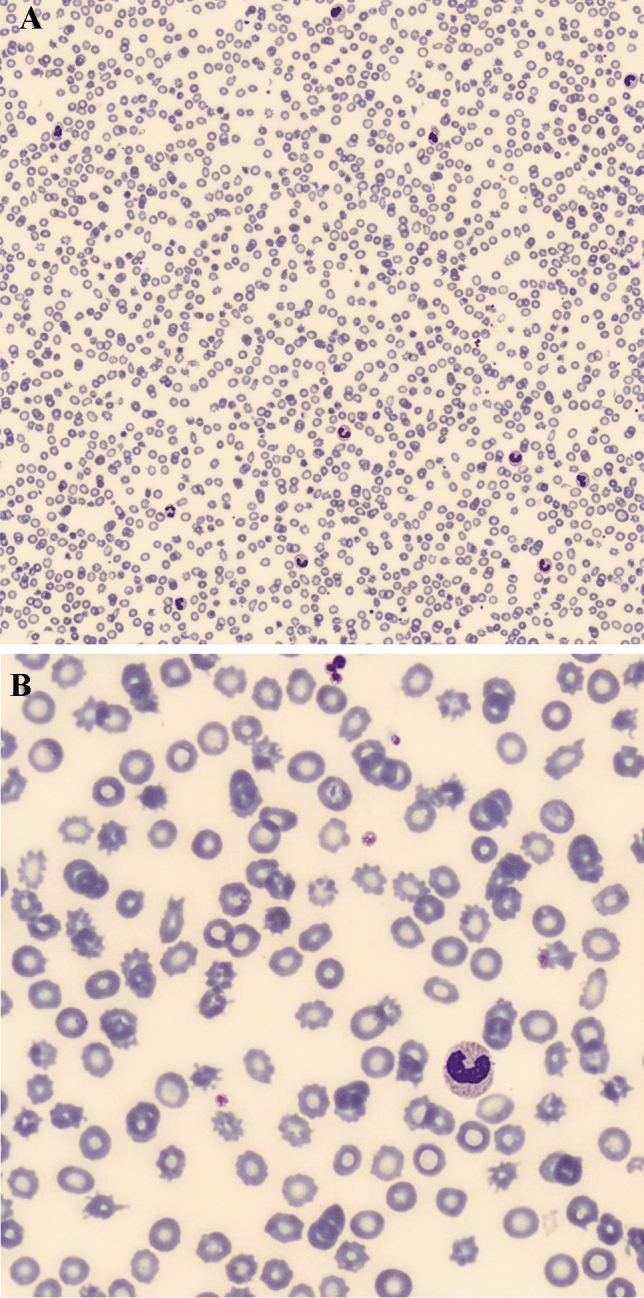
**A** Peripheral blood smear from the cirrhotic patient, showing spur cells, i.e., red blood cells with spike-like projections. **B** Peripheral blood smear at a higher magnification

Laboratory tests were highly suspicious for non-immune and non-microangiopathic haemolytic anaemia because of elevated unconjugated bilirubin, undetectable haptoglobin, reactive reticulocytes count, stable platelets and negative direct/indirect Coombs tests. Since peripheral blood smear (performed when haemoglobin was 5.8 g/dL) revealed a high number (> 20%) of acanthocytes, echinocytes, dacrocytes and cellular fragments (Fig. 1), we hypothesized spur cell anaemia. The diagnosis was corroborated by the characteristic lipid profile, with very low total cholesterol, ApoA1 and ApoB, the clinical context and the absence of probable alternatives.

During hospitalization, the patient required numerous RBC transfusions to obtain a target of at least 7.5 g/dL haemoglobin. However, before discharge, the patient showed decreased unconjugated bilirubin and stable haemoglobin concentration over 3–5 days without transfusions; a peripheral blood smear was repeated and confirmed the presence of spur cells (22% spur cells).

In consideration of the severity of alcoholic chronic disease, age and comorbidity, the patient was unfortunately not suitable for liver transplantation and died few weeks later of acute hepatic decompensation.

*Prospective study*

## Methods

### Study design and data collection

Patients involved in this single center, prospective study were referred to our Liver Unit, and consecutively enrolled from September 2020 to May 2021. The study was approved by the Ethical Committee of Verona (CESC2524). All subjects signed a written informed consent. Inclusion criteria were: a diagnosis of liver cirrhosis of any etiology (alcoholic, hepatitis C and B, autoimmune/primary biliary cholangitis (PBC), metabolic) and functional class. Exclusion criteria were the following: hepatocellular carcinoma and other malignancies, bone marrow diseases (both benign and malignant disorders), nutritional deficiencies (iron, folates, vitamin B12), heart failure and advanced chronic kidney disease. Presence of at least moderate ascites, gastro-esophageal varices within last six months, hepatic encephalopathy defined as at least 1 episode of grade 1 or more of West-Haven scale in the last 6 month, were recorded. Child–Pugh (CP) score and Model for End-Stage Liver Disease (MELD) score were calculated at time of enrollment. A blood smear was analyzed semi-automatically to estimate the percentage of acanthocytes and echinocytes. In each patient, a blood sample was drown to obtain a complete blood count with mean corpuscular volume (MCV) and relative distribution width (RDW), total and fractionated bilirubin, haptoglobin, albumin, International Normalized Ratio (INR), creatinine, sodium, ferritin, folates and vitamin B12. One year liver-related mortality (due to acute decompensation or ACLF) was reported with survival time from initial assessment. ACLF diagnosis was made according to the latest European Foundation for the Study of Chronic Liver Disease (EF-CLIF)[16]. Anaemia was defined as haemoglobin less than 13 and 12 g/dL in male and non-pregnant females, respectively (WHO definition) [17], while a cutoff of 8 g/dL was considered for diagnosing severe anaemia in both sexes [18].

Since a cutoff of 5% spur cells (acanthocytes plus echinocytes) is considered to be relevant for clinical purposes [12], patients were sub-grouped according to the amount of spur cells in smear between > 5%, 1–5% and < 1%.

## Statistical analysis

Continuous variables were expressed as mean ± standard deviation (SD) and as median with Inter-Quartile Range (IQR) depending on the data distribution; discrete variables were expressed as number and percentage. Comparison between continuous variables was performed using the Student’s *T* test and/or the Mann–Whitney *U* test, depending on the distribution of the data. Pearson’s chi square test was performed to assess differences between frequencies in groups. The risk of one-year liver-related mortality was tested using Cox-Regression analysis and Kaplan–Meier method. Log-rank test was used to compare patients with > 5% spur cells in blood smear with others. Univariate COX-Regression analyses were performed to assess potential factors associated with ACLF development during follow-up and 1 year liver-related mortality (age, gender, severe anaemia, CP score, MELD score and spur cells > 5 or 1–5%). Variables that resulted significantly associated with the outcome in univariate analyses (*p* < 0.05) or considered clinically relevant were entered into the stepwise multivariate model.

A statistically significant value was considered if a *p* < 0.05. SPSS Statistics 22 was used for all data analysis.

## Results

One-hundred and nineteen consecutive patients (median age 67 [IQR 58–73], males 73.9%) were enrolled. Alcoholic etiology of cirrhosis was diagnosed in 43.7% of subjects, HCV/HBV in 35.7%, autoimmune/PBC in 10.1%, metabolic in 10.9%. The majority of patients was CP class-B (42.7%), while 37.5% and 19.6% were in class A and C, respectively.

Eleven out of 119 patients (9.2%) had > 5% spur cells in blood smear while 24.4% (29/119) had 1–5%. Among those with > 5% spur cells, 7 out of 11 had alcoholic cirrhosis and 2 had clinical evidence of low-grade active haemolysis. 8 out of 11 patients died during follow-up and only 1 patient had LT for end-stage liver disease.

### Subgroups analysis

Among the included patients, 3 groups were defined according to the number of spur cells,

 < 1%, 1–5% and > 5%. In patients with > 5% spur cells in blood smear, haemoglobin and haptoglobin were lower compared with those with < 1%, while CP score, MELD score, albumin, INR, ferritin, creatinine and unconjugated bilirubin were higher, with no differences in folates, vitamin B12, ammonia and total bilirubin (Table 2). Comparisons of variables between the other sub-groups (> 5% vs. 1–5% vs < 1%) are shown in Table 2. A large proportion of patients had anaemia, especially patients having more spur cells (Table 2); however, the prevalence of severe anaemia was not significantly different between patients with > 5% spur cells (2 out of 11, 18,2%) and those with < 1 and 1–5% spur cells (9 out of 118, 8,3%; *p* = 0.269). No clinical or biochemical factors were found to be predictive of spur cells percentage in blood smear.

**Table 2:** Comparison between sub-groups, identified upon percent of spur cells in the blood smear

	< 1%, *n*° 79	1–5%, *n*° 29	> 5%, *n*° 11
Age, years	65 [58–70]	70 [56–78]	70 [61–73]
Gender, males (%)	70	83	82
Alcoholic aetiology (%, n°)	39.2 (31)	48.2 (14)	63.6 (7)
Hb, g/dL	12.1 [10.6–13.8] §	10.4 [8.7–12.3]	9.7 [9.0–11.1] §
MCV, fL	97 [91–104]	97 [92–101]	98 [91–102]
RDW, %	15 [14–17]	16 [14–17]	18 [15–19]
Platelets, /mm3	118 [61–152]	100 [65–131]	91 [49–150]
INR	1.2 [1.0–1,4] §	1.2 [1.1–1.4]	1.5[1.2–1.8] §
Albumin, g/L	34 [29–38] §	32 [27–37]	27 [24–34] §
Total bilirubin, mg/dL	1.0 [0.6–2.1]	1.6 [1.1–2.5]	1.9 [1.3–2.8]
Unconjugated bilirubin, mg/dL	0.5 [0.3–1.4]	0.7 [0.4–1.0]	0.9 [0.4–1.5]
Haptoglobin, g/L	1.0 [0.5–1.4] §	0.8 [0.7–0.9]	0.3 [0.1–0.9] §
CP score	7.0 [6.0–8.0]	7.5 [5–10]	9.0 [8.0–12.0]
MELD	11 [8–15] §	14 [9–16] *	18 [14–20] *§
Anaemia (%)	48.1§	65.5*	90.9*§
Ferritin, ng/mL	314 [141–769] §	267 [114–734] *	859 [249–1359] *§
Folates, ng/mL	5.6 [4.0–11.2]	6.3 [4.7–20.1]	5.6 [4.0–11.3]
Vitamin B12, mcg/dL	517 [403–857]	600 [500–1400]	997 [700–1470]
Serum creatinin, mg/dL	0.80 [0.67–1.20] §	1.05 [0.77–1.26]	1.23 [0.98–1.40] §
Ascites (%, *n*°)	50.0 (37) §	70.8 (17) *	100.0 (11) §*
Varices (%, *n*°)	56.3 (40) §	46.4 (13) *	90.9 (10) §*
HE (%, *n*°)	20.1 (11) §	23.1 (6) *	63.6 (7) §*
Liver-related death (%, *n*°)	17.7 (14) §	34.5 (10) *	72.7 (8) §*

*Hb* haemoglobin, *MCV* mean corpuscular volume, *RDW* red-cells distribution width, *INR* International normalized ratio, *CP* Child–pugh score, *MELD* Model for end stage Liver Disease, Anaemia, haemoglobin < 13 g/dL for male, < 12 g/dL for female, *HE* Hepatic Encephalopathy

^*^means 1–5% vs. ≥ 5%

^§^ means > 5% vs. < 1%; in all *p* < 0.05

Patients with > 5% spur cells were significantly more often decompensated compared with all other sub-groups (< 1%, 1–5%). Almost all patients with more than 5% spur cells developed ACLF (3 grade 1, 3 grade 2 and 2 grade 3) during follow-up, significantly differing from those with less spur cells (8 out of 11, 72.7% vs. 31 out of 108, 28.7%, *p* = 0.02). Subjects with > 5% spur cells had significantly higher 1 year liver-related mortality compared with patients with 1–5% and < 1%, as shown in Table 2.

In multivariate logistic regression analysis, the presence of > 5% spur cells was significantly associated only with CP score (OR 1.74 95% I.C. [1.17–2.58], *p* = 0.006).

In multivariate Cox-regression analysis, CP score and spur cells > 5%, but not severe anaemia, were independently related to ACLF development during follow-up (Table 3).

**Table 3:** Hazard Ratios (HR) for ACLF development and 1 year liver-related mortality according to predisposing risk factors. Fully adjusted values by the use of Cox-regression analysis

	ACLF development fully adjusted HR (95% I.C.)	*p* value	1 year liver-related mortality Fully adjusted HR (95% I.C.)	*p* value
Spur cells > 5%	3.43 [1.46–8.01]	< 0.001	3.30 [1.40–7.80]	< 0.001
CP score	1.42 [1.18–1.73]	< 0.001	1.51 [1.22–1.86]	< 0.001
Age (years)			1.07 [1.02–1.11]	0.002
MELD score			1.02 [1.01–1.03]	0,021

*CP* Child–pugh score, *MELD* Model for end stage Liver Disease

**Fig. 2:**
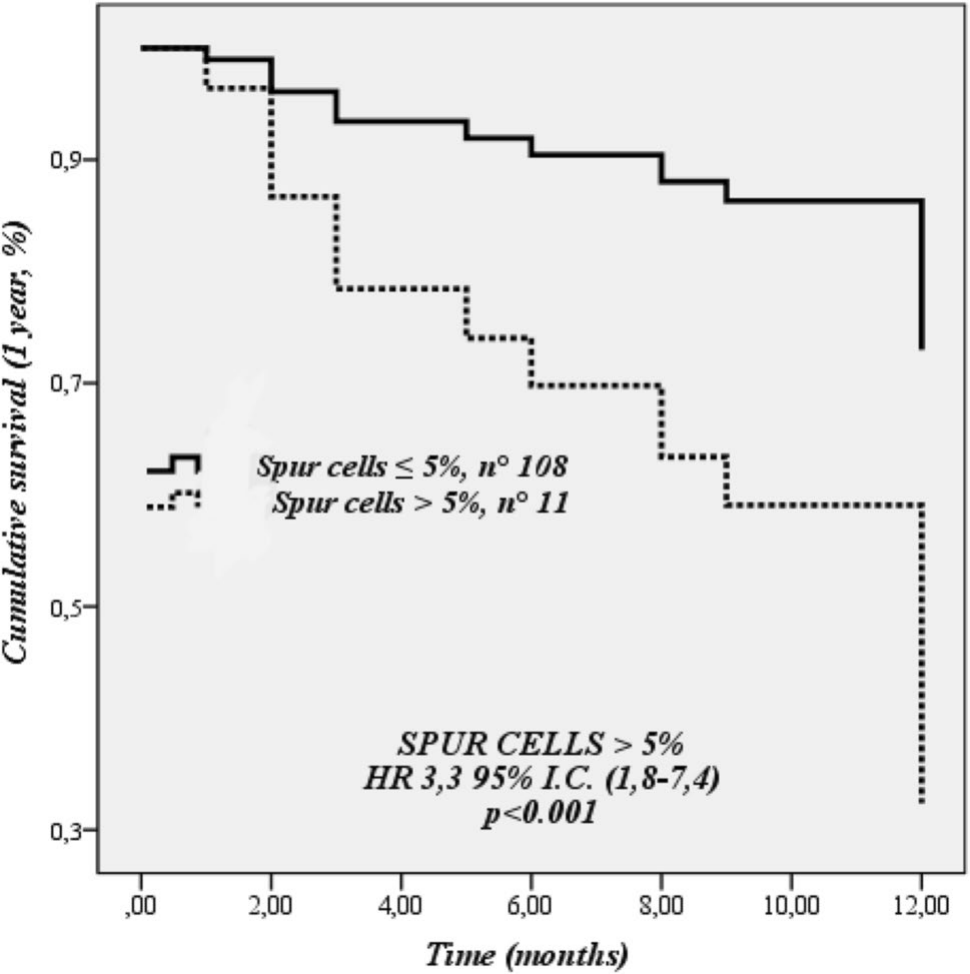
Cox-regression analysis for cumulative survival at 1 year (liver-related death)

Similarly, age, MELD score, CP score and the presence of > 5% spur cells were independently associated with 1 year liver-related mortality (Table 3, Fig. 2); after exclusion of patients with > 5%, only age (HR 1.06 95% I.C.[1.01–1.10], *p* = 0.016), CP score (HR 1.53 95% I.C.[1.22–1.92], *p* < 0.001 and MELD score (HR 1.02 95% I.C.[1.01–1.04], *p* = 0.034), but not the presence of 1–5% spur cells, were independently related to liver-related mortality.

## Discussion

The results of this prospective study showed that 9.2% of outpatient cirrhotic patients had “spur cell anaemia”, i.e., > 5% spur cells in blood smear. The vast majority of them had alcoholic-related cirrhosis and almost none had clinically overt haemolysis. Spur cells anaemia was strongly and independently associated with early ACLF development and liver-related mortality. Altogether, this is the first report showing that even without haemolysis and severe anaemia, the presence of spur cells in blood smear is a critical finding that should urge prevention of ACLF and quick pre-LT assessment.

Spur cell anaemia is an uncommon haemolytic non-immune and non-microangiopathic anaemia which typically occurs in patients with advanced liver disease, especially, but not exclusively [12, 19], of alcoholic aetiology [20]. Typically, patients present severe coagulopathy, severe anaemia and marked icterus. The most distinguishing characteristic is probably the presence, in blood smear, of a variable percentage of spiky-cells [21, 22]. These dysmorphic erythrocytes have numerous projections emerging from the cellular membrane that irreparably reduce the stability and the flexibility of the cells throughout the bloodstream probably determining a premature phagocytosis or spontaneous rupture in splenic sinusoids.

This peculiar modification seems not to be related to a defective erythropoiesis but is an acquired disorder of RBC occurring in circulating blood, mainly due to a pathological modification of lipid composition [23] and defective phospholipids metabolism [24]. In fact, both free cholesterol and the ratio between cholesterol and phospholipids in all plasma lipoproteins were shown to be increased in “spur cells” patients despite a characteristic severe total hypocholesterolaemia and markedly reduced level of low-density lipoprotein (LDL). Recent evidences underline the role of low concentrations of Apo-AII in determining an imbalance between high-density lipoproteins sub-fractions (increased high-density lipoprotein HDL-2, reduced HDL-3) potentially leading to impaired lipid trafficking throughout RBC [10, 23]. Furthermore, cholesterol and phosphatidylcholine highly-rich erythrocytes with a spur-cell pattern were found in subjects with extra-hepatic biliary obstruction and hyperbilirubinemia and this was shown to be reversible after resolution of cholestasis [25]. On the other hand, spur cells were shown to develop after incubation of healthy RBC with plasma from subjects with spur cells, proving a role of circulating mediators of lipid metabolism [26]. Recent evidences hypothesized a bidirectional link between an altered iron metabolism with parenchymal overload (hyperferritinemia due to alcohol-related intestinal hyper-absorption and haemolysis, markedly impaired hepcidin production due to persistent inflammation and reduced hepatic biosynthesis) and the severity of liver disease with possible haemolysis [27, 28]; in fact, spur cell haemolysis could be the culprit cause of iron overload found in several explanted livers after LT for alcoholic cirrhosis [29]. Another interesting feature of spur cell anaemia is its relationship with prolonged prothrombin time. First of all, a higher INR is expected in patients with more severe liver disease, but it is thought that the phospholipid alteration occurring in spur cell anaemia can alter the intrinsic and extrinsic pathway of clotting [15] causing prolongation of both INR and activated partial thromboplastin time (aPTT).

Spur cell anaemia is relatively rare and the prevalence among cirrhotic patients is largely unknown and probably under-diagnosed. However some authors reported a prevalence between 16.7% [12] and 31% [11] in patients with decompensated cirrhosis. Since clinically significant haemolysis was found when spur cells were > 5%, this has been included in a recent report as the diagnostic criterion [12]. However, the presence of spur cells is reported to be common in liver disease [11, 12], suggesting a high prevalence as “sub-clinical” feature. In contrast, a clinically overt expression was found to be infrequent, but associated with poor prognosis and high mortality [11]. As regards ACLF, only a series of 5 severely decompensated subjects was described to have spur cell anaemia [30]; in particular, a clear direct relationship was found between the percentage of spur cells and all parameters related to liver disease severity (MELD score and its components) and poor survival [11, 12].

Anecdotal experiences with plasmapheresis [31], antioxidants [32], high-dose steroids [33] and lipid-sequestering resins [10, 32] were reported to have some beneficial effects in reducing the need for RBC transfusions but no effects on survival. In fact, apart from sporadic reports of conservatively treated patients [34], only LT was shown to be effective in reversing spur cell haemolysis and overall survival [14, 34].

When compared with other studies [11, 12], we showed a relatively high prevalence of spur cells in blood smear of patients with liver cirrhosis, especially considering that in our cohort all CP classes were well represented while other authors considered the prevalence only among decompensated subjects [11, 12]. In particular, even without an overt haemolysis, almost 10% of our outpatient subjects had > 5% spur red blood cells. In our cohort, it was associated with alcoholic aetiology and this is in line with previous studies. As expected, patients with > 5% spur cells, compared with subjects with < 1%, had lower haemoglobin and albumin, higher CP score, MELD score, serum creatinine, ferritin, INR and unconjugated bilirubin but similar values of MCV, RDW and platelets. Almost all of them developed ACLF during follow-up further demonstrating that spur cells represent a feature associated with worse prognosis. They were significantly more decompensated because of ascites, hepatic encephalopathy and high-risk varices. Interestingly, ferritin and serum creatinine were higher in spur cells patients. Despite lower clinical strength, similar results were obtained also in patients with fewer spur cells (1–5%). This could be a further proof of the fact that detecting this altered RBC is an early and sub-clinical marker of disease severity.

To date, this is the first report showing spur cells to be independently related with ACLF. In fact, at survival analysis, the presence of spur cells > 5%, but not 1–5%, was strongly and independently associated with ACLF and liver-related mortality, together with age, CP score and MELD score, although with lower statistical strength. This confirms that the cutoff of 5% is clearly associated with increased short-term complications and liver-related mortality and is in line with previous reports and studies in which a few-months survival was reported for patients with > 5% spur cells [10]. The presence of 1–5% spur cells was not associated with increased mortality, similarly to what reported by Malik at all [36], but at least in our study, with a more severe and decompensated liver disease.

Previous studies have shown that anaemia is highly prevalent in cirrhosis [37, 38] and our data are in line with them as a progressive decrease of haemoglobin level has been shown as liver function deteriorates and the prevalence of spur cells increases. Moreover, anaemia has been shown elsewhere to be a risk factor for ACLF [38] and short term survival [37]. Unlike those studies, in our cohort baseline severe anaemia (i.e., haemoglobin < 8 g/dL) per se was not found as an independent predictor of outcome and this could be related to the fact that only anaemia, as considered in its general meaning, but not that related to spur cells, has been considered.

In our cohort, differently from previously published studies, the vast majority of patients with > 5% spur cells (9 out of 11) didn’t have severe anaemia at enrolment and did not require blood transfusions during follow-up. This confirms that only spur cells, but not the anaemia itself, are clinically relevant in predicting ACLF and mortality. In this context, spur cells in cirrhosis should not be systematically associated with the term anaemia but re-defined as *spur cell disease*, a specific feature with a strict and strong association with poor prognosis.

Our study has some limitations. First of all, the small number of the study population and the high prevalence of alcoholic etiology of liver disease. Secondly, a deep insight on the molecular pathogenesis of the disease is lacking. Furthermore, a confirmatory independent cohort is lacking, as this could draw a firm conclusion.

In conclusion, spur cells in liver cirrhosis are not a rare finding, even in the outpatient setting. The cutoff 5% spur cells, also when unrelated to haemolytic anaemia, represents a critical independent risk factor for the development of early liver-related complications, such as ACLF, and death. Consequently, we suggest to perform a blood smear in all cirrhotic patients with or without anaemia, and especially in those with decompensated diseases, very high ferritin levels and, obviously, anaemia.

As the current assessment for prioritizing patients for LT is based on MELD score, which does not consider the presence of spur cells, larger studies are needed to solve the unmet need to expand the classical MELD-based indications for LT and standardize in clinical practice the role of spur cells to better stratify the risk of death and thus calling for prompt medical intensive care management.

## Abbreviations

WHO: World health organization
CP score: Child–Pugh score
MELD score: Model for end-stage Liver Disease score
INR: International normalized ratio
LT: Liver transplantation
ACLF: Acute on Chronic Liver Failure
